# SNVSniffer: an integrated caller for germline and somatic single-nucleotide and indel mutations

**DOI:** 10.1186/s12918-016-0300-5

**Published:** 2016-08-01

**Authors:** Yongchao Liu, Martin Loewer, Srinivas Aluru, Bertil Schmidt

**Affiliations:** 1 School of Computational Science & Engineering, Georgia Institute of Technology, Atlanta, 30332 Georgia USA; 2 Translational Oncology, Johannes Gutenberg University Medical Center gGmbH Mainz, Mainz, 55131 Germany; 3 Institute of Computer Science, Johannes Gutenberg University Mainz, Mainz, 55128 Germany

**Keywords:** SNP calling, Somatic SNV calling, Bayesian model, Indel calling

## Abstract

**Background:**

Various approaches to calling single-nucleotide variants (SNVs) or insertion-or-deletion (indel) mutations have been developed based on next-generation sequencing (NGS). However, most of them are dedicated to a particular type of mutation, e.g. germline SNVs in normal cells, somatic SNVs in cancer/tumor cells, or indels only. In the literature, efficient and integrated callers for both germline and somatic SNVs/indels have not yet been extensively investigated.

**Results:**

We present SNVSniffer, an efficient and integrated caller identifying both germline and somatic SNVs/indels from NGS data. In this algorithm, we propose the use of Bayesian probabilistic models to identify SNVs and investigate a multiple ungapped alignment approach to call indels. For germline variant calling, we model allele counts per site to follow a multinomial conditional distribution. For somatic variant calling, we rely on paired tumor-normal pairs from identical individuals and introduce a hybrid subtraction and joint sample analysis approach by modeling tumor-normal allele counts per site to follow a joint multinomial conditional distribution. A comprehensive performance evaluation has been conducted using a diversity of variant calling benchmarks. For germline variant calling, SNVSniffer demonstrates highly competitive accuracy with superior speed in comparison with the state-of-the-art FaSD, GATK and SAMtools. For somatic variant calling, our algorithm achieves comparable or even better accuracy, at fast speed, than the leading VarScan2, SomaticSniper, JointSNVMix2 and MuTect.

**Conclusions:**

SNVSniffers demonstrates the feasibility to develop integrated solutions to fast and efficient identification of germline and somatic variants. Nonetheless, accurate discovery of genetic variations is critical yet challenging, and still requires substantially more research efforts being devoted. SNVSniffer and synthetic samples are publicly available at http://snvsniffer.sourceforge.net.

## Background

Next generation sequencing (NGS) technologies provide affordable, reliable and high-throughput sequencing of DNA, and make it possible to comprehensively catalog genetic variations in human genomes. Single-nucleotide variation is one of the most common genetic variations in human individuals. The single-nucleotide variants can be further interpreted as germline SNVs, i.e. single-nucleotide polymorphisms (SNPs), in normal cells or somatic SNVs in cancer/tumor cells. Up to date, a variety of computational methods have been developed to call germline or somatic SNVs from NGS read data and a typical pileline based on NGS comprises: (*i*) sequence read quality control (e.g. read error correction and duplicate removal); (*ii*) align sequence reads from one or more samples to the genome using leading aligners (e.g. [1–4]); (*iii*) realign reads around indels to facilitate indel calling; (*iv*) call variants using probabilistic methods (e.g. Bayesian model); and (*v*) assess the statistical significance of the called variants and report the results. Note that some methods also call indels along with SNVs.

A number of single-sample SNV callers have been developed for NGS, and representative callers include MAQ [5], SOAPsnp [6], SAMtools [7], SNVMix [8], GATK [9], and FaSD [10]. MAQ, SOAPsnp and FaSD model allele counts at each site as a binomial distribution, while SNVMix uses a mixed binomial distribution. All of the four callers identify SNVs by computing Bayesian-based posterior probabilities. Both SAMtools and GATK employ Bayesian likelihood and provide support for the processing of pooled data. It should be noted that these SNV callers actually can be applied to identify any single-nucleotide genetic variation in an individual, including both germline and somatic variants, albeit originally targeting SNPs. In addition, some of these tools do not call indels. Refer to [11] for more details about the state-of-the-art research on genotyping and single-sample SNV calling.

Compared to germline SNV calling, somatic SNV calling is more challenging since its objective is to identify alleles that appear in the tumor, but do not occur in the host’s germ line. In other words, we have to additionally distinguish germline polymorphisms from somatic ones at the sites containing variants. One approach [8] is to first call SNVs in the tumor using conventional SNP callers and then screen the predicted SNVs against public SNP databases, e.g. dbSNP [12]. Unfortunately, this approach is challenged by the considerable number of novel SNVs found in individuals, e.g. [13] reported that 10 ∼50 % of SNVs per individual are novel events. In this case, germline mutations uncatalogued in public databases would be falsely identified as somatic mutations.

A more reliable approach to detecting somatic mutations is to call variants in both a tumor sample and its matched normal sample. Approaches used by existing somatic SNV callers can be classified into two categories: simple subtraction and joint sample analysis. The simple subtraction approach separately genotypes the normal and tumor samples at each site and then classifies the site as somatic if the genotype in the normal is homozygous reference and the genotype in the tumor contains alternative alleles to the reference base. This also suggests that callers based on simple subtraction can directly use well-established single-sample SNV callers such as SAMtools and GATK. This simple subtraction approach may provide reasonable prediction for sample pairs with high somatic allele frequency and data purity. However, it has been observed that somatic mutations are prevalent at a low frequency in clinical samples [14]. In this case, any tendency to mistake germline mutations for somatic ones may potentially contaminate the discovery of somatic SNVs. On the other hand, there are variations in somatic allele frequencies from site to site or sample to sample, which are often caused by substantial admixture of normal cells in the tumor sample, copy number variations and tumor heterogeneity. In this regard, a joint analysis of both samples is expected to be capable of further improving performance, by facilitating simultaneous tests for alleles in both samples and enabling more comprehensive representation of tumor impurity and noisy data. Several somatic SNV callers have been developed based on joint sample analysis, including VarScan2 [15], SomaticSniper [16], JointSNVMix2 (JSM2) [17], Strelka [18], MuTect [19] and FaSD-somatic [20]. Albeit employing a simple subtraction approach at the core, VarScan2 pioneered to jointly evaluate the statistical significance of allele frequency information in tumor-normal samples. SomaticSniper, JSM2, Strelka, MuTect and FaSD-somatic all employ Bayesian models to jointly analyze the tumor-normal pair, while adopting diverse specific procedures or formulas. In addition, unlike other somatic callers that only focus on SNV calling, VarScan2 provides additional support for somatic indel calling.

In this paper, we present SNVSniffer, an integrated solution to fast and efficient identification of both germline and somatic SNVs/indels. This algorithm relies on genotype inference using Bayesian probabilistic models to identify SNVs, and investigated a multiple ungapped alignment (MUA) approach to call indels. For germline variant calling, at each site we model its allele count vector to follow a multinomial conditional distribution, and then single out the most likely genotype by computing Bayesian posterior probabilities. For somatic variant calling, we use paired tumor-normal samples from identical individuals, and at each matched site we consider the allele count vector in the normal to be a mixture of reference bases, diploid germline variants or artificial bases (e.g. from sequencing cycles or alignment process), and the allele count vector in the tumor to be a mixture of bases from normal cells and somatic variants besides artificial bases. Moreover, we investigate a hybrid somatic SNV calling approach by combing a subtraction analysis with a joint sample analysis, where joint sample analysis models the joint allele count vector from the tumor-normal pair to follow a joint multinormal distribution. For performance comparison, we have used the SMASH [21] and GCAT [22] benchmarks for germline variant calling, and have used synthetic tumors from simulated data, virtual tumors [19] from real sequencing data, and real mouse and human tumors for somatic variant calling. Through our evaluations, in terms of germline variant calling, SNVSniffer demonstrates highly competitive accuracy and faster speed than the top-performing FaSD, GATK and SAMtools algorithms. Meanwhile, in terms of somatic variant calling, our algorithm achieves comparable or even better accuracy compared to the leading VarScan2, SomaticSniper, JSM2 and MuTect algorithms, while demonstrating highly competitive speed.

## Results and discussion

The assessment of SNVSniffer (v2.0.4) is conducted from two aspects: single-sample germline variant calling and somatic variant calling from tumor-normal pairs. For germline variant calling, we have used SMASH [21], a benchmarking toolkit for human genome variant calling and GCAT [22], a genome comparison and analytic testing platform for optimizing variant discovery from personal genomes. For somatic variant calling, we have generated synthetic tumors from simulated and real data, respectively, and also used real tumors acquired from the Cancer Genome Atlas (TCGA) and TrON Mainz (Germany) [23]. For synthetic data, recall, precision and *F*-score are used to measure performance, because of the known ground truth. Recall is defined as $\frac {TP}{TP+FN}$, precision as $\frac {TP}{TP+FP}$ and *F*-score as $\frac {2\times Recall\times Precision}{Recall + Precision}$, where TP is the number of true positives, FP is the number of false positives and FN is the number of false negatives. For real datasets, sensitivity and specificity are used. Sensitivity and specificity is defined as $\frac {TP}{TP+FN}$ and $\frac {TN}{TN+FP}$, respectively, where TN is the number of true negatives.

In this paper, unless otherwise specified, all tests are conducted on a workstation with two Intel Xeon X5650 2.67 GHz hex-core CPUs and 96 GB RAM, running the Linux operating system (Ubuntu 14.04). Likewise, the runtime is measured in wall clock time by default and every caller runs in sequential. Both VarScan2 and FaSD take mpileup-formatted input files, while other callers all use BAM-formatted inputs. For SNVSniffer, we have implemented three execution modes. The first mode (M1) directly applies our calling engine to BAM-formatted inputs. The second mode (M2) realigns the reads, whose alignments have indels or soft clipped ends, to calculate per-base alignment quality (BAQ) scores [24] and then inputs the new alignments to our calling engine. The third mode (M3) re-aligns all reads to calculate BAQ scores as SAMtools does. This realignment procedure could improve overall calling quality, but at the cost of lower speed and the potential loss of sensitivity, as per our experiences. In addition, GATK used the accurate “HaplotypeCaller” subprogram for variant calling.

It needs to be stressed that the FaSD executable binary (source code is not publicly available) encountered an Illegal Instruction error on the aforementioned workstation. Fortunately, we managed to execute FaSD in another personal computer (PC) with an Intel i7-4770 quad-core 3.4 GHz CPU and 16 GB memory, running the Ubuntu 14.04 operating system. Since FaSD was executed sequentially, its speed could be considered directly proportional to the core frequency of the CPU used. In other words, it is reasonable to estimate the actual runtime of FaSD on the workstation by multiplying its runtime on the PC by a constant factor 1.273 (i.e. 3.40 GHz ÷ 2.67 GHz).

### Germline variant calling

#### SMASH benchmarks

We first evaluated our caller using the SMASH benchmark toolkit and then compared it to three leading germline variant callers including SAMtools (v1.3), GATK (v3.5) and FaSD (latest version). In this evaluation, two types of benchmarks in SMASH are used, namely the synthetic benchmark and the sampled human benchmark. The synthetic benchmark comprises two read datasets: Venter and Contaminated Venter, both of which are derived from the Craig Venter’s genome (HuRef) with the variants provided by [25]. The sampled human benchmark consists of four read datasets: NA12878, contaminated NA12878 (denoted as NA12878+ in our context), NA18507 and NA19240. The NA12878 dataset is derived from a European female (NA12878), the NA18507 dataset from a Nigerian male (NA18507) and the NA19240 dataset from a Nigerian female (NA19240). Moreover, the NA12878+ dataset is obtained by contaminating the NA12878 dataset with reads from the NA12878 individual’s husband (NA12877). For these benchmarking datasets, SMASH released the alignments files in addition to raw sequence reads. In this regard, we did not realign the reads in each benchmarking dataset, and instead directly used the ready-to-use alignments. In addition, in the consideration of speed, our study merely used the reads all aligned to the human chromosome 20. Tables 1 and 2 show the performance comparison using the synthetic benchmark and the sampled human benchmark, respectively.

**Table 1:** Performance and runtimes on SMASH synthetic benchmark

Caller	SNP calling (%)			Indel calling (%)			Time(s)
	Recall	Precision	*F*-score	Recall	Precision	*F*-score	
Venter							
SNVSniffer(M1)	**98.5**	97.1	97.8	68.9	83.0	75.3	**133**
SNVSniffer(M2)	98.3	97.4	97.8	69.0	83.7	75.6	320
SNVSniffer(M3)	97.9	98.5	98.2	70.4	83.6	76.4	1331
SAMtools	98.3	97.1	97.7	63.7	69.5	66.5	2046
GATK	98.1	**99.1**	**98.6**	**76.2**	**86.2**	**80.9**	2538
FaSD	98.0	97.5	97.7	−	−	−	2005
Contaminated venter							
SNVSniffer(M1)	98.0	97.4	97.7	69.0	84.0	75.8	**119**
SNVSniffer(M2)	97.8	97.8	97.8	68.7	84.5	75.8	336
SNVSniffer(M3)	97.3	**98.7**	**98.0**	69.7	84.3	76.3	1387
SAMtools	**98.1**	97.3	97.7	62.7	72.1	67.1	2046
GATK	97.9	96.8	97.3	**75.8**	**86.4**	**80.8**	2803
FaSD	97.4	97.5	97.4	−	−	−	2070

Best results are highlighted in boldface

**Table 2:** Performance and runtimes on SMASH sampled human benchmark

Caller	NA12878	NA12878+	NA18507	NA19240
Sensitivity (%)				
SNVSniffer(M1)	98.9	98.9	99.0	99.1
SNVSniffer(M2)	98.9	98.9	98.9	99.0
SNVSniffer(M3)	98.8	98.8	98.9	99.0
SAMtools	**99.2**	**99.2**	99.3	99.4
GATK	99.1	99.1	**99.3**	**99.5**
FaSD	98.9	98.9	99.1	99.2
Time(s)				
SNVSniffer(M1)	**226**	**203**	**190**	**206**
SNVSniffer(M2)	560	541	474	1065
SNVSniffer(M3)	2550	2543	2093	3379
SAMtools	3730	3694	3147	3379
GATK	6541	6249	6321	5936
FaSD	2132	2054	1979	2068

Best results are highlighted in boldface

##### On Venter synthetic dataset

For SNP calling, the recall is 98.5 % for SNVSniffer(M1), 98.3 % for SNVSniffer(M2) and 97.9 % for SNVSniffer(M3), suggesting that more broad application of BAQ score [24] to reads could result in decreased recall. On the contrary, precision gets improved as the execution mode moves from M1 (precision 97.1 %) via M2 (precision 97.4 %) to M3 (precision 98.5 %). These two observations are consistent with our expectations as mentioned above. *F*-score has a roughly consistent trend with precision, where the value is 97.8 % for M0, 97.8 % for M2 and 98.2 % for M3. In terms of recall, SNVSniffer(M1) performs best, while SNVSniffer(M2) and SAMtools are jointly second best. In terms of precision, GATK is best with 99.1 % precision and is immediately followed by SNVSniffer(M3) with 98.5 % precision. Both SNVSniffer(M1) and SAMtools yield the worst precision. In terms of *F*-score, GATK is best and SNVSniffer(M3) second best. Meanwhile, both SAMtools and FaSD perform worst. For indel calling, GATK performs best with respect to all measures and SAMtools the worst. The recall is 76.2 % for GATK and 63.7 % for SAMtools; the precision is 86.2 % for GATK and 69.5 % for SAMtools; and the *F*-score is 80.9 % for GATK and 66.5 % for SAMtools. SNVSniffer(M3) yields the second best recall of 70.4 % and the second best *F*-score of 76.4 %, while SNVSniffer(M2) gave the second best precision of 83.6 %.

##### On Contaminated Venter synthetic dataset

Due to the contaminated nature of this dataset, the performance ranking between callers becomes different compared to the Venter dataset. For SNP calling, similar to the Venter dataset, SNVSniffer yields decreasing recall, increasing precision and increasing *F*-score as the execution mode moves from M1 via M2 to M3. More specifically, the recall is 98.0 % for M1, 97.8 % for M2 and 97.3 % for M3; the precision is 97.4 % for M1, 97.8 % for M2 and 98.7 % for M3; and the *F*-score is 97.7 % for M1, 97.8 % for M2 and 98.0 % for M3. SAMtools achieves the best recall of 98.1 %, while SNVSniffer(M1) performs second best. SNVSniffer(M3) yields the best precision and *F*-score, whereas GATK has the worst precision of 96.8 % and *F*-score of 97.3 %. For indel calling, GATK achieves the best recall of 95.8 %, the best precision of 86.4 % an the best *F*-score of 80.8 %. In addition, SAMtools performs worst for each measure.

##### On sampled human datasets

In this evaluation, we used sensitivity to measure the performance of a caller. SNVSniffer(M1) and SNVSniffer(M2) achieve ≥89.9 *%* sensitivity for each dataset. The average sensitivity is 99.0 % for M1, 98.9 % for M2 and 98.9 % for M3. SAMtools achieves the best sensitivity for the NA12878 and NA12878+ datasets, while GATK performs best for the rest. On average, the sensitivity is 99.3 % for SAMtools, 99.3 % for GATK and 99.0 % for FaSD.

##### Speed comparison

For each benchmarking dataset, SNVSniffer(M1) is undoubtedly the fastest caller. On the Venter dataset, this caller achieves a speedup of 15.3 over SAMtools, a speedup of 19.0 over GATK and a speedup of 15.0 over FaSD (estimated actual speedup of 19.1). On the Contaminated Venter data, it achieves higher speedups over each of the other callers. Concretely, the speedup is 17.2 over SAMtools, 23.5 over GATK and 17.4 over FaSD (estimated actual speedup of 22.2). On the sample human benchmark, SNVSniffer(M1) runs up to 18.2× faster than SAMtools, up to 33.3× faster than GATK and up to 10.4× faster than FaSD (estimated actual speedup of 13.2). Even though SNVSniffer(M2) and SNVSniffer(M3) are slower than SNVSniffer(M1), they are still considerably faster than SAMtools, GATK and FaSD for each benchmarking dataset.

#### GCAT benchmark

The GCAT platform provides a variant calling test, which uses the sequencing data from the NA12878 human individual to evaluate germline variant callers. An Illumina paired-end read datatset is used in this study. This dataset is generated from the exome capture of NA12878 and has 150× coverage. All reads in this dataset are aligned using BWA (v0.7.5a) to get the initial alignments. For the sake of indel calling, the initial alignments are further processed by the IndelRealigner subprogram in GATK (v3.5) which locally realigns the reads around indels. As per our experiences, this realignment procedure does facilitate performance improvement for variant calling. To assess variant calling quality, GCAT uses the Genome in a Bottle (GIAB) [26] high-confidence calls as the gold standard. GIAB targets the well-studied NA12878 individual and is produced by integrating different sequencing platforms, read aligners and variant callers [22]. Note that in this test, FaSD continued to be executed in the PC as mentioned above.

Table 3 shows the performance comparison using the GCAT benchmark. For SNP calling, SAMtools achieves the best sensitivity of 97.57 % and the best specificity of 99.9989 %. As for *T*_*i*_/*T*_*v*_ (the ratio of transition to transversion in SNP), its value is expected to be around 2.8 for whole human exome sequencing [22]. Hence, for *T*_*i*_/*T*_*v*_ in whole human exome sequencing, the closer to 2.8 the better calling quality. This is because the presence of false positive mutations will drop the overall mean closer to 0.5 (the theoretical value if there is no molecular bias). In this regard, SNVSniffer(M3) performs best with *T*_*i*_/*T*_*v*_=2.251, while SNVSniffer(M1) and SNVSniffer(M2) are second best and third best, respectively. GATK is superior to SAMtools, while FaSD is the worst. For indel calling, GATK performs best by yielding a sensitivity of 95.28 % and a specificity of 99.9997 %. SAMtools performs worst in terms of sensitivity and SNVSniffer worst in terms of specificity. As for speed, SNVSniffer(M1) is fastest and achieves a speedup of 16.7 over SAMtools, a speedup of 21.6 over GATK and a speedup of 23.5 over FaSD (estimated actual speedup of 29.9). For modes M2 and M3, albeit not as fast as mode M1, their speed is still significantly superior to SAMtools, GATK and FaSD.

**Table 3:** Performance and runtimes on GCAT Illumina 150× exome sequencing data

Caller	SNP calling (%)			Indel calling (%)		Time(s)
	Sensitivity	Specificity	*T*_*i*_/*T*_*v*_	Sensitivity	Specificity	
SNVSniffer(M1)	94.86	99.9982	2.223	50.48	99.9981	**766**
SNVSniffer(M2)	94.86	99.9983	2.235	50.94	99.9981	931
SNVSniffer(M3)	94.69	99.9982	**2.251**	50.94	99.9981	7597
SAMtools	**97.57**	**99.9989**	1.450	43.08	99.9987	12825
GATK	97.31	99.9982	1.920	**95.28**	**99.9997**	16568
FaSD	79.83	99.9922	1.123	−	−	17986

Best results are highlighted in boldface

### Somatic variant calling

We evaluated the somatic variant calling performance of SNVSniffer using synthetic tumors from simulated data, virtual tumors from real data and real tumors. This performance was further compared to four selected leading somatic variant callers, i.e. VarScan2 (v2.3.7), SomaticSniffer (v1.0.4), JSM2 (v0.8-b2) and MuTect (v1.1.4). Among these callers, JSM2 outputs the probabilities of joint genotypes rather than explicitly report somatic mutations as other callers do. In this regard, as suggested by the authors [17], the probability of a site being a somatic location is computed as *P*(*AA*,*AB*)+*P*(*AA*,*BB*). In our evaluations, a somatic site is deemed to be valid as long as its probability is ≥0.9 for JSM2. Moreover, as shown in germline variant calling, SNVSniffer(M1) demonstrates highly competitive calling quality compared to the leading callers including SAMtools, GATK and FaSD, while achieving superior speed. In this regard, we will merely use SNVSniffer(M1) for somatic variant calling performance comparison in the following. In addition, for somatic variant calling, the runtime of SNVSniffer counts in the execution time spent on the estimation of tumor purity, where tumor purity represents the expected percentage of reads coming from tumor cells. The tumor purity estimation procedure is input-dependent and can take half of the overall runtime at maximum. If the value of tumor purity is specified at start-up, the tumor purity estimation procedure will not be conducted, thus significantly improving speed. Details about our tumor purity estimation approach can be obtained from [27].

#### On synthetic tumors from simulated data

We have simulated three tumor-normal sample pairs from the human chromosome 21 (UCSC hg38) with uniform base sequencing error rate 1.0 %, 1.5 % and 2.0 % respectively (refer to [27] for more details about the simulation). Each sample is comprised of 100-bp Illumina-like paired-end reads with a mean insert size of 500 and 30× coverage over the reference. For each tumor, we have set the expected tumor purity to 0.9, the fraction of indels among mutations to 0.15 and the probability of indel extension to 0.3 for the simulation. For each sample, we aligned all reads using BWA (v0.7.5a) to get the initial alignments and employed the GATK IndelRealigner subprogram to further process them, considering the existence of indel mutations.

Table 4 shows the performance comparison. In terms of recall, SNVSniffer(M1) outperforms any other caller for each dataset, by achieving a recall of 94.86 % for error rate 1.0 %, 94.49 % for error rate 1.5 % and 94.00 % for error rate 2.0 %. MuTect performs second best and is immediately followed by JSM2 and SomaticSniper in decedent order of ranking. VarScan2 is inferior to any other caller. In terms of precision, SNVSniffer(M1) outperforms both VarScan2 and MuTect for all datasets, by producing a precision of 95.87 %, 95.86 % and 95.85 % for error rate 1.0 %, 1.5 % and 2.0 %, respectively. SomaticSniper performs best, while MuTect is worst. In terms of *F*-score, SNVSniffer(M1) performs best for error rate 1.0 % and JSM2 second best. In contrast, for the remaining two error rates, JSM2 takes the first place and our caller the second place. For each dataset, VarScan2 performs worst while SomaticSniper outperforms VarScan2 and MuTect. In terms of speed, our caller does not outrun SomaticSniper for each dataset, because of tumor purity estimation embedded in our caller. Nevertheless, our caller demonstrates significantly faster speed than VarScan2, JSM2 and MuTect. On average, our caller runs 7.0× faster than VarScan2, 5.0× faster than JSM2 and 13.4× faster than MuTect. MuTect is the slowest caller, while JSM2 runs faster than VanScan2.

**Table 4:** Somatic SNV calling performance comparison

Metric	Error	SNVSniffer(M1)	SomaticSniper	VarScan2	JSM2	MuTect
Recall	1.0 %	**94.86**	86.62	77.43	91.26	93.47
	1.5 %	**94.49**	86.62	80.67	91.24	93.47
	2.0 %	**94.00**	86.62	80.50	91.23	93.47
Precision	1.0 %	95.87	**99.77**	95.02	99.68	84.76
	1.5 %	95.86	**99.77**	95.29	99.68	84.76
	2.0 %	95.85	**99.78**	95.36	99.68	84.76
*F*-score	1.0 %	**95.36**	92.73	85.33	95.28	88.90
	1.5 %	95.17	92.73	87.37	**95.27**	88.90
	2.0 %	94.92	92.74	87.30	**95.27**	88.90
Time(s)	1.0 %	275	**183**	1828	1364	3677
	1.5 %	273	**180**	1947	1361	3644
	2.0 %	271	**179**	1990	1368	3658

Best results are highlighted in boldface

Since only our caller and VarScan2 support indel calling, we have further compared both callers in terms of indel calling (see Table 5). From the table, it can be seen that the recall, precision and *F*-score is relatively low for either caller. SNVSniffer(M1) is superior to VarScan2 for each dataset with respect to every metric. Concretely, our caller yields 30.20 % recall, 24.35 % precision and 26.96 % *F*-score for error rate 1.0 %, 30.20$ recall, 24.35 % precision and 26.96 % *F*-score for error rate 1.5 % and 30.20 % recall, 24.36 % precision and 26.97 % *F*-score for error rate 2.0 %.

**Table 5:** Somatic indel calling performance comparison

Error	Caller	Recall	Precision	*F*-score
1.0 %	SNVSniffer(M1)	**30.20**	**24.35**	**26.96**
	VarScan2	14.91	17.71	16.19
1.5 %	SNVSniffer(M1)	**30.20**	**24.35**	**26.96**
	VarScan2	15.26	17.53	16.32
2.0 %	SNVSniffer(M1)	**30.20**	**24.36**	**26.97**
	VarScan2	14.83	17.22	15.94

Best results are highlighted in boldface

#### On virtual tumors from real data

We have used virtual tumors [19] to assess the performance of somatic SNV callers. Virtual tumors only contain somatic SNVs and are produced from real sequence reads of two human individuals by following the procedure described in [19]. We have generated 10 virtual tumors with tumor purity uniformly ranging from 0.1 to 1.0, and implanted 4,436 somatic SNV mutations. At each somatic mutation site, the normal genotype is homozygous reference while the tumor genotype is heterozygous reference accordingly.

**Fig. 1:**
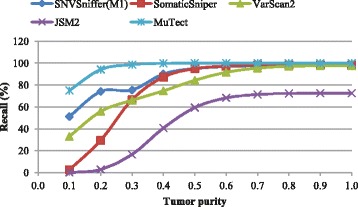
Recall on virtual tumors in the function of tumor purity

**Fig. 2:**
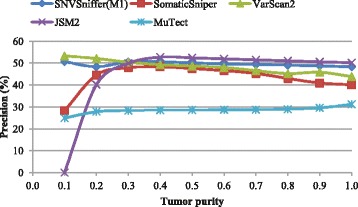
Precision on virtual tumors in the function of tumor purity

**Fig. 3:**
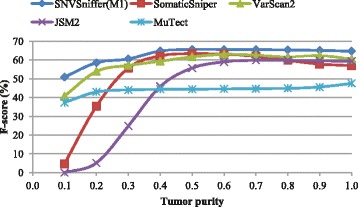
*F*-score on virtual tumors in the function of tumor purity

Figures 1, 2 and 3 show the recall, precision and *F*-score as a function of tumor purity, respectively. For each virtual tumor, our caller yields the best *F*-score and MuTect the best recall. In terms of recall, our caller always outperforms VarScan2 and JSM2. In comparison with SomaticSniper, our caller is superior for the virtual tumors with purity ≤0.5, while the former performs better for the rest. It should be noted that MuTect managed to identify all somatic sites for the virtual tumors with purity ≥0.7, but none of all other callers is able to make it. In terms of precision, VarScan2 is best for the two virtual tumors with purity 0.1 and 0.2, respectively. SNVSniffer is best for the virtual tumor with purity 0.3 and JSM2 best for the rest. Meanwhile, both SomaticSniper and MuTect are inferior to our caller. In terms of *F*-score, JSM2 always yields the worst performance. SomaticSniper outperforms VarScan2 for the two virtual tumors with purity 0.4 and 0.5, while the latter is superior for the rest.

#### On real tumors

##### Sensitivity assessment

To measure sensitivity, we have used 5 whole genome sequencing tumor-normal pairs for the Ovarian serous cystadenocarcinoma disease, all of which are obtained from the TCGA project. The accession identifiers are TCGA-13-0885-01A-02W-0421-09 (T1), TCGA-13-1481-01A-01W-0549-09 (T2), TCGA-13-1488-01A-01W-0549-09 (T3), TCGA-24-1417-01A-01W-0549-09 (T4), and TCGA-24-1424-01A-01W-0549-09 (T5), respectively. The gold-standard somatic variants used here are in part based on the data generated by the TCGA Research network (http://cancergenome.nih.gov). In this test, we have executed each caller on a supercomputer with each node equipped with 4 AMD Opteron 6272 CPUs of 2.1 GHz frequency and 16 cores. Moreover, we have measured the runtime in CPU time, instead of wall clock time as used before, in order to evade the impact of job scheduling on the supercomputer.

Table 6 shows the sensitivity and runtime comparison. For each tumor, MuTect achieves the best sensitivity (>90 *%* each). SNVSniffer(M1) yields the second best sensitivity (>66 *%* each) for all tumors with an exception that on tumor T3, SomaticSniper outperforms ours by a small margin. SNVSniffer(M1) and SomaticSniper (>61 *%* sensitivity each) are always superior to VarScan2 (>35 *%* sensitivity each). Interestingly, JSM2 does not succeed in identifying any true variant for each case. In terms of speed, SomaticSniper runs fastest and SNVSniffer(M1) second fastest (note that tumor purity estimation took about half of the runtimes for our caller). Nevertheless, our algorithm is still considerably faster than all other callers and achieves an average speedup of 1.85 over VarScan2, 3.39 over JSM2 and 7.91 over MuTect.

**Table 6:** Sensitivity and runtimes comparison using real tumors

Dataset	SNVSniffer(M1)	VarScan2	SomaticSniper	JSM2	MuTect
Sensitivity (%)					
T1	66.86	38.29	61.148	0.001	**91.43**
T2	82.35	35.29	63.73	0.00	**93.14**
T3	72.36	59.35	75.61	0.00	**95.93**
T4	93.55	83.87	87.10	0.00	**96.77**
T5	75.00	43.75	68.75	0.00	**97.92**
Time (h)					
T1	2.67	5.75	1.58	10.41	22.87
T2	2.56	4.73	1.33	9.48	19.37
T3	2.41	4.30	1.34	7.39	19.27
T4	2.91	5.26	1.39	10.00	24.16
T5	2.87	4.75	1.55	8.11	20.50

Best results are highlighted in boldface

##### Specificity assessment

Assuming alignments are correct, we can identify any site with at least one non-reference read as a mutation candidate. Obviously, this very aggressive approach can lead to the development of an extremely sensitive variant caller, but will also result in enormous false positives [19]. Therefore, given a variant caller, characterizing its specificity becomes critical in evaluating its calling accuracy using real tumors. Since we are not aware of the ground truth of somatic variants in real tumors, one approach to measuring specificity is (*i*) first producing two read datasets from an identical tumor sample by two separate sequencing experiments and (*ii*) then considering both datasets as a tumor-normal pair and input them to somatic variant callers. In this way, the ideal number of true positives is zero and all mutations identified are necessarily false positives.

In this test, we have used two real exome sequencing datasets generated from two separate sequencing experiments of an identical epithelial mouse tumor, i.e. the CT26 colon carcinoma cell line studied in TrON Mainz (Germany) [23]. Both datasets are sequenced using an Illumia HiSeq 2000 sequencer and are also publicly available at European Nucleotide Archive (ENA) under the accession numbers ERR424934 and ERR424935, respectively. The alignments are gained by exactly following the data processing procedure described in [23]. Given a caller, we input the ERR424934 alignments as the normal and the ERR424935 alignments as the tumor, and then execute the caller on the workstation mentioned above. Table 7 shows the specificity (only SNVs are taken into account) and runtime comparison for all callers. From the table, none of the evaluated callers achieves zero false positive, suggesting the difficulty in accurate somatic variant calling in some sense. Concretely, VarScan2 yields 4507 false positives and therefore achieves the best specificity of 99.9998 %. SNVSniffer(M1) produces 12,387 false positives and therefore performs second best with a specificity of 99.9995 %. MuTect performs worst with the most 2,463,700 false positives and the smallest specificity of 99.9096 %. JSM2 is superior to SomaticSniper, where the specificity is 99.9986 % for the former and 99.9923 % for the latter. As for speed, SomaticSniper runs fastest and SNVSniffer(M1) second best. Nonetheless, in comparison with all others our caller demonstrates considerably faster speed with a speedup of 1.6 over VarScan2, 2.8 over JSM2 and 4.8 over MuTect.

**Table 7:** Specificity and runtime comparison using real tumors

Caller	FP	Specificity (%)	Time (h)
SNVSniffer(M1)	12387	99.9995	1.8
SomaticSniper	209530	99.9923	**1.2**
VarScan2	**4507**	**99.9998**	2.9
JSM2	38550	99.9986	5.0
MuTect	2463700	99.9096	8.7

Best results are highlighted in boldface

## Conclusion

Advances in NGS technologies have enabled us to conduct genome-wide identification and cataloging of genetic variations in a cost-effective manner. In this paper, we have presented SNVSniffer to provide a fast, efficient and integrated calling algorithm for both germline and somatic single-nucleotide and indel mutations. For SNV calling, Bayesian models are the core of our algorithm. Although Bayesian models are frequently used in variant calling, an integrated solution to both germline and somatic variant discovery has not yet been extensively investigated in the literature. Technically, in terms of germline SNV calling we model allele counts per site to follow a multinomial distribution and employ a Bayesian model to infer the most likely genotypes per site and then determine variants via genotype interpretation. On the other hand, in terms of somatic SNV calling we model the paired tumor-normal allele count to follow a joint multinomial distribution, and then investigate a hybrid approach that combines subtraction analysis with a joint sample analysis to infer genotypes for both samples.

We have conducted a comprehensive study to evaluate the performance of our algorithm and then compare this performance to existing state-of-the-art callers. For germline variant calling, SNVSniffer achieves highly competitive accuracy at superior speed, compared to the leading SAMtoosl, GATK and FaSD algorithms. For somatic variant calling, SNVSniffer achieves comparable or better accuracy than the selected top-performing SomaticSniper, VarScan2, JSM2 and MuTect algorithms, while demonstrating highly competitive speed. In particular, firstly, performance evaluation using synthetic tumors showed that SNVSniffer performs best in terms of recall, associated with relatively high precision and *F*-score for each tumor. In contrast, MuTect performs worst in terms of both precision and *F*-score. Secondly, performance evaluation using virtual tumors demonstrated that SNVSniffer always achieves the best *F*-score, while holding relatively high precision. In contrast, MuTect has the best recall but along with the worst precision. Thirdly, performance evaluation on real tumors exposed that MuTect and SNVSniffer yield the best and second best sensitivity, respectively, while VarScan2 and SNVSniffer performs best and second best with respect to specificity. Finally, SNVSniffer has superior speed to VarScan2, JSM2 and MuTect, albeit slightly slower than SomaticSniper. Nonetheless, for somatic variant calling, there are still some limitations and challenges. Firstly, the normal sample is assumed to be an admixture of germline mutations and noise. This assumption does not always hold since contamination may occur in normal cells. Secondly, the accuracy of somatic indel calling is still relatively low based on our evaluations. Thirdly, our caller does not take into account some more complex genomic variations in cancer such as copy number variations and sub-clonal populations. How to address such limitations and challenges is part of our future work. As the sequencing of matched tumor-normal samples is becoming a popular routine in cancer research, we still demand more accurate yet efficient calling algorithms for somatic variants at practical levels of tumor purity.

## Methods

**Fig. 4:**
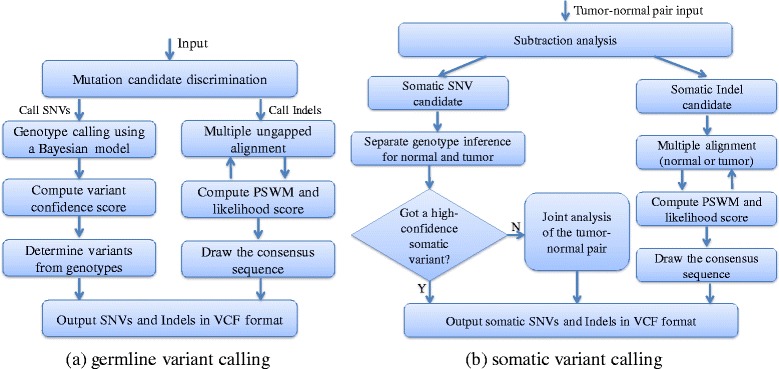
Program diagram of SNVSniffer for variant calling

SNVSniffers supports for the discovery of SNVs and indels. For SNVs, our algorithm identifies them based on genotype inference from Bayesian posterior probabilities. For indels, our algorithm relies on accurate alignment of indels to the reference and employs a MUA approach to derive consensus sequences for indels called. Moreover, our algorithm accepts three file formats: pileup (from MAQ), mpileup (from SAMtools) and BAM [7] (default setting). In our previous study [27], we used SNVSniffer version 1.0 and this version does not natively support BAM. In this case, in order to use BMA-formatted inputs, we need to launch a separate SAMtools child process at start-up to perform conversion from BAM to mpileup at the runtime. In this study, however, we used the enhanced SNVSniffer version 2.0, and this version has enabled native support for BAM. Therefore, we do not need SAMtools to perform on-the-fly format conversion any more. Figure 4 illustrates the program diagram of SNVSniffer for germline and somatic variant calling.

### Germline variant calling

#### SNV calling

We model allele counts at each site of the genome as a multinomial distribution conditioned on genotypes. At site *i*, we define *X*_*i*_ to denote the allele count vector, *X*_*i*,*j*_ to denote the aligned allele over *Σ*={A, C, G, T} from read *j*, *γ* to denote the reference base, and $G_{k}={G_{k}^{1}}{G_{k}^{2}} \in $ {AA, CC, GG, TT, AC, AG, AT, CG, CT, GT} to denote a genotype for diploid genomes (1≤*k*≤*K* and *K* is the total number of genotypes, i.e. 10 in our case). In our caller, the probability *P*(*X*_*i*_|*G*_*k*_) of observing allele count vector *X*_*i*_ at site *i* is defined as

1 $$  P(X_{i}|G_{k})\propto \prod\limits_{j=1}^{N}\prod\limits_{c\in\Sigma}P(X_{i,j}|G_{k})^{I(X_{i,j}=c)}  $$

where *N* is the number of alleles covering the site and *I*(*X*_*j*_=*x*) is an indicator function whose value is 1 if *X*_*i*,*j*_ equals *x* and 0 otherwise. For each aligned allele *X*_*i*,*j*_, the probability *P*(*X*_*i*,*j*_|*G*_*k*_) of observing this allele at the site is defined as

2 $$  P(X_{i,j}|G_{k})=\alpha P(X_{i,j}|{G_{k}^{1}}) + (1 - \alpha) P\left(X_{i,j}|{G_{k}^{2}}\right)  $$

by taking into account a few factors such as sequencing bias between distinct haploid chromosomes, base-calling errors and alignment quality.

In Eq. (2), *α* denotes the proportion of reads sequenced from the ${G_{k}^{1}}$ haploid chromosome, and is set to 0.5 in our implementation based on the assumption that the two haploid chromosomes are impartially sequenced. In practice, this assumption may not always be the case since we have observed the existence of strand distribution bias in real sequencing data. In this regard, we have ever attempted a sliding-window-based approach to compute site-specific *α* values. Given a site and a sliding window size (e.g. 1000), the value of *α* at the site is computed by averaging the per-site percentage of bases aligned to the forward strand of the reference in the sliding window centering at the site. Considering that it cannot differentiate which strand ${G_{k}^{1}}$ corresponds to in Eq. (2), we re-formulate Eq. (2) as

3 $$  P\left(X_{i,j}|G_{k}\right)=\max\left\{ \begin{array}{ll} \alpha P\left(X_{i,j}|{G_{k}^{1}}\right) + \left(1 - \alpha\right) P\left(X_{i,j}|{G_{k}^{2}}\right)\\ \left(1 - \alpha\right) P\left(X_{i,j}|{G_{k}^{1}}\right) + \alpha P\left(X_{i,j}|{G_{k}^{2}}\right)\\ \end{array} \right.  $$

Unfortunately, we did not notice obvious performance change after using this equation in comparison with Eq. (2) through our limited evaluations (results are not reported here). Therefore, we have continued to use Eq. (2) in our caller. Nevertheless, it may be interesting to investigate the possibility of employing site-specific strand distribution information to improve calling quality.

In Eq. (2), $P\left (X_{i,j} | {G_{k}^{b}}\right)$ means the probability of observing *X*_*i*,*j*_ at the ${G_{k}^{b}}$ haploid chromosome (*b* = 1 or 2), and is defined as

4 $$  P\left(X_{i,j}|{G_{k}^{b}}\right) = \left\{ \begin{array}{ll} \omega_{i,j}& \text{if} X_{i,j} = {G_{k}^{b}}\\ \left(1 - \omega_{i,j}\right)\Phi\left(X_{i,j}, {G_{k}^{b}}\right)& \text{otherwise}\\ \end{array} \right.  $$

where *Φ*(·) is a 2-dimensional probability table, with $\Phi (X_{i,j}, {G_{k}^{b}})$ representing the probability of ${G_{k}^{b}}$ being the true chromosomal base given that *X*_*i*,*j*_ is miscalled or misaligned, and *ω*_*i*,*j*_ is the accuracy (or weight) of *X*_*i*,*j*_. This equation is inspired by [9], but has two major differences. On one hand, [9] classifies alleles covering each site as whether reference base or non-reference variant, and thereby partitions the full set of genotypes over three genotype categories: homozygous reference, heterozygous reference and homozygous variant. On the contrary, SNVSniffer does not perform such allele classifications. Instead, we consider the total of *K* possible genotypes, which can also be further classified into four categories: homozygous reference, heterozygous reference, homozygous variant and heterozygous variant. On the other hand, mapping quality scores are additionally introduced to our computation. In SNVSniffer, *ω*_*i*,*j*_ is calculated as

5 $$ \omega_{i,j} = 1 - \frac{2\times 10^{-0.1\left(B_{q} + M_{q}\right)}}{10^{-0.1 B_{q}} + 10^{-0.1 M_{q}}}  $$

where *B*_*q*_ is the base quality score and *M*_*q*_ is the mapping quality score.

As for $\Phi \left (X_{i,j}, {G_{k}^{b}}\right)$, a naïve approach is to use non-informative prior, i.e. set $\Phi \left (X_{i,j}, {G_{k}^{b}}\right)$ to 1/(|*Σ*|−1) for each allele *X*_*i*,*j*_ that is not equal to ${G_{k}^{b}}$. Alternatively, we can also inspect the error profiles of different sequencing technologies and then derive *Φ*(·) for use. In SNVSniffer, we have used the probabilities for Illumina sequencing given in [9].

##### Genotype inference

Having gained *P*(*X*_*i*_|*G*_*k*_) for each genotype *G*_*k*_, we compute the posterior probability *P*(*G*_*k*_|*X*_*i*_) of the true genotype being *G*_*k*_ given *X*_*i*_, based on the Bayes’ theorem, where *P*(*G*_*k*_|*X*_*i*_) is computed as

6 $$  P(G_{k}|X_{i}) = \frac{P(X_{i}|G_{k})P(G_{k})}{\sum\limits_{l=1}^{K} P(X_{i}|G_{l})P(G_{l})}\propto P(X_{i}|G_{k})P(G_{k})  $$

Subsequently, we single out the genotype with the largest posterior probability as the "true" genotype at the site. In general, we need to show that the larger probability of the selected genotype is statistically significant compared to the others. You et al. [28] proposed the use of Dixon’s *Q* test [29] which originally targets the detection of outliers. The *Q* test examines the ratio of the absolute difference between the largest and the second largest numbers, to the range of evaluated numbers, and then compute a *P*-value at a specific confidence level to guide whether to reject or accept the hypothesis. In our algorithm, we have attempted to use the *Q* test to evaluate the statistical significance of the most likely genotype. However, through our evaluations we did not find any significant differences in the performance of genotype calling, when compared to the case without using the *Q* test. Considering the computational overhead of this *Q* test, we have disabled this test, and instead directly select the genotype with the largest probability.

##### Genotype priors computation

In Eq. (6), we require a prior probability for each genotype *G*_*k*_. In our algorithm, we have considered three implementations of prior probabilities: non-informative priors, priors derived from heterozygous mutation rate *θ* [16], and priors derived from both *θ* and transition/transversion (*T*_*i*_/*T*_*v*_) ratio [20]. Specifically, *θ* means the expected rate of heterozygous point mutations in the population of interest and its estimated value is close to 10^−3^ between two distinct human haploid chromosomes [6]. *T*_*i*_/*T*_*v*_ ratio is around 2.0 ∼2.1 for whole human genome sequencing as shown in the recent human genome studies, particularly the 1000 genomes project [13]. For non-informative priors, each genotype is assumed to have the same prior probability $\frac {1}{K}$. For the *θ*-only priors, they are defined as

7 $$  {} P(\!G_{k}\!)\,=\,\!\left\{\!\! \begin{array}{ll} \theta& \text{if \(G_{k}\) is heterozygous reference} \\ \theta^{2}& \text{if \(G_{k}\) is heteozygous variant} \\ \theta / 2& \text{if \(G_{k}\) is homozygous variant} \\ 1 \,-\, \sum\limits_{l=1}^{K} P(G_{l})I(G_{l})& \text{if \(G_{k}\) is homozygous reference} \end{array} \right.  $$

where *I*(*G*_*l*_) returns 0 if *G*_*l*_ is homozygous reference, and 1, otherwise.

For the *T*_*i*_/*T*_*v*_-based priors, they are similarly defined to Eq. (7), but additionally check whether the genotype *G*_*k*_ has a transition or transversion mutation relative to the reference base at each site. Intuitively, more accurate results can be yielded if the priors used are consistent with the ground truth, and otherwise, misleading results may be caused by the use of unrealistic priors. Through our evaluations, it is interesting to find that none of the three priors is able to consistently show superior performance. In this regard, we have chosen the *θ*-only priors as the default setting, since it has been more often observed to have better performance in our limited number of tests. In addition, we have pre-computed the priors for every combination of reference bases with genotypes in order to improve speed.

##### Variant confidence score

To trade off sensitivity and specificity, we have introduced a variant confidence score (VCOS) to measure our confidence of the correctness of the variants called. VCOS is only computed for the inferred genotypes that are not homozygous reference, i.e. genotypes must have ≥1 alternative allele to *γ*.

8 $$  VCOS = \frac{-\sum\limits_{j=1}^{N} I(X_{i,j}\in G_{k})\cdot \log(P(X_{i,j}|\gamma))}{\sum\limits_{j=1}^{N} I(X_{i,j}\in G_{k})}  $$

where *I*(*X*_*i*,*j*_∈*G*_*k*_) equals 1 if *X*_*i*,*j*_ is an allele in the inferred genotype *G*_*k*_, and 0, otherwise. *P*(*X*_*i*,*j*_|*γ*) means the probability of substituting the reference base *γ* for the aligned allele *X*_*i*,*j*_, and is computed as

9 $$  P(X_{i,j}|\gamma) = \left\{ \begin{array}{ll} S_{ti}& \text{if \(X_{i,j}\) is a transition of \(\gamma\)} \\ S_{tv}& \text{if \(X_{i,j}\) is a transversion of \(\gamma\)} \\ 1 - \theta& \text{if \(X_{i,j} = \gamma\)} \end{array} \right.  $$

by incorporating heterozygous mutation rate *θ* and the *T*_*i*_/*T*_*v*_ ratio *δ* in the population of interest. In Eq. (9), *S*_*ti*_=*δθ*/(1+*δ*) and *S*_*tv*_=0.5*θ*/(1+*δ*). We have set *δ* to 2.0 for human samples.

Based on VCOS, our caller classifies the variants called into three categories: high-confidence, low-confidence and false positives, depending on how many alternative alleles to *γ* are there in the corresponding genotypes. A variant is deemed as high-confidence if its VCOS is ≥*HC*(*G*_*k*_), as low-confidence if its VCOS is <*HC*(*G*_*k*_) but ≥*LC*(*G*_*k*_), and as false positives, otherwise. The score threshold *HC*(*G*_*k*_) is computed as

10 $$ HC(G_{k}) = \left\{ \begin{array}{ll} -\frac{1}{2}\log((1 - \theta)\times S_{ti})& \text{Case 1}\\ -\log(S_{ti})& \text{Case 2} \end{array} \right.  $$

and *LC*(*G*_*k*_) computed as

11 $$ {}LC(G_{k})\!\! =\!\! \left\{\!\/1 \begin{array}{ll} -\phi\log(1 \,-\, \theta)-\frac{1}{2}(1 \,-\, \phi)\log(S_{ti}\!\times\! S_{tv})& \text{Case 1}\\ -\frac{1}{2}\psi\log(S_{ti}\times S_{tv}) \,-\, (1 \,-\, \psi)\log(1\! -\! \theta)& \text{Case 2}\\ \end{array} \right.  $$

where Case 1 means that *G*_*k*_ is heterozygous reference and Case 2 means that *G*_*k*_ does not contain *γ*. Note that we have constrained the values of *ϕ* and *ψ* to ensure that *HC*(*G*_*k*_) is always ≥*LC*(*G*_*k*_).

#### Indel calling

Compared to SNV calling, indel calling based on NGS read alignment is more challenging, thus having motivated the development of a few dedicated indel callers (e.g. [30–33]). In general, these challenges comes from the following aspects. On one hand, the existence of indels will interfere with alignment quality, though state-of-the-art aligners yield fully gapped alignments. On the other hand, due to local similarity nature between reads and the reference, dynamic-programming-based semi-global and local alignment are widely used for NGS read alignment, rather than global alignment. In this case, indels may result in (*i*) alignments with soft clipping at the ends and (*ii*) multiple optimal alignment paths per read to the same reference region (implementation dependent). The former would cause indels to be out of our sight in the alignment map. The latter may result in wrong selection of correct alignment path for a given read, thus causing to miss the correct indel location and even incur artificial presence of indels. This is because these alignment paths may have distinct distributions of point insertions or deletions. In [32], it is believed that an approach that combines de novo genome assembly and genome-genome alignment is most powerful. However, this approach is expected to have prohibitive and daunting computational cost and also subject to assembly and alignment quality of available state-of-the-art tools to some extent.

##### Lightweight indel candidate identification

In this paper, we propose a lightweight indel candidate identification approach by assuming alignments are correct. In this case, realignment of reads around indels becomes an important procedure before applying our approach to call indels. In our approach, we assume that if a site is an indel candidate, there is at least one indel operation on this site relative to the reference. After finding such a site, we conduct two statistical examinations using Fisher’s exact test in order to remove bias caused by sequencing and alignment in practice. The first test computes if the number of variant alleles is statistically significant compared to the number of references bases at this site under a certain mutation rate. If significant, this site will not be considered as an indel candidate; otherwise, we continue to the second test. The second test computes if the number of indel operations is statistically significant compared to the number of references bases at this site. If significant, this site is deemed as an indel candidate and otherwise, we discard this site. In practice, this lightweight approach not only leads to fast speed, but also demonstrates highly competitive indel calling accuracy compared to the leading SAMtools and GATK through our limited evaluations (refer to the “Results and discussion” section).

##### Multiple ungapped alignment

Given an indel mutation candidate, a set of sequences *S*={ *S*_0_, *S*_1_, …, *S*_*n*−1_} (defined over the alphabet *Σ*) will be observed from the alignment map and each sequence represents the inserted or deleted consecutive bases relative to the reference. Moreover, these sequences may contain different consecutive bases, due to reasons such as sequencing errors and alignment bias. In practice, this is usually the case based on our observations from real data. In this regard, we have investigated a MUA approach with the intention to find the *most significant* consensus representation of a set of sequences of varied lengths possibly.

Our MUA approach is inspired by the starting point search procedure of the MEME motif discovery algorithm [34, 35]. Define *l*_*min*_ and *l*_*max*_ to denote the minimum and maximum sequence length in *S*, respectively, and *S*_*i*,*j*,*l*_ to denote the substring of *S*_*i*_ that starts at position *j* and contains *l* characters. Our MUA approach can be described as follows. Given a specific *l* (*l*_*min*_≤*l*≤*l*_*max*_), any *l*-length substring in *S* is deemed as a seed. Starting from a seed *S*_*i*,*j*,*l*_, we align it by not allowing gaps to every sequence *S*_*u*_ (0≤*u*<|*S*|) and subsequently single out the substring *S*_*u*,*v*,*l*_ with the largest alignment score for each *S*_*u*_. This set of highest-scoring substrings forms a MUA and is subsequently used to calculate a position-specific weight matrix (PSWM) of size |*Σ*|×*l*. Having got the PSWM, we compute a likelihood score for the MUA and then use this score to select the best consensus representation of *S*. Algorithm 1 shows the pseudocode of our MUA approach.



Given a MUA, its PSWM is computed by associating it with a background frequency specification for each character in *Σ*. For the convenience of discussion, we assume that characters in *Σ* have already been encoded to distinct integers in the range [ 0,|*Σ*|). For a PSWM *W*, its element *W*_*i*,*j*_ (0≤*i*<|*Σ*| and 0≤*j*<*l*) is defined as

12 $$  W_{i,j} = \log_{2}\left(\frac{p_{i,j}}{q_{i}}\right)  $$

In this equation, *p*_*i*,*j*_ denotes the site-specific frequency of character *i* at column *j* and is computed by dividing the number of occurrences of character *i* at column *j* by |*S*|. *q*_*i*_ represents the background frequency of character *i* and is computed as $\frac {1}{|\Sigma |}$ in our caller by default.

##### Likelihood score computation

Given a MUA of length *l* columns, we can draw a consensus sequence *S*^′^ from the alignment by first computing a PSWM using Eq. (12) and then deriving *S*^′^ from the PSWM by selecting the character with the largest weight within each column. To measure the significance of *S*^′^, we compute a likelihood score to indicate the probability of observing *S*^′^. Letting *S*^′^[ *i*] denote the *i*-th character of *S*^′^ (0≤*i*<|*S*^′^|), the likelihood score for *S*^′^ is calculated as

13 $$  L=\frac{1}{l}\log_{2}\left(\sum\limits_{j=0}^{l-1} W_{S'[j],j}\right)  $$

In this case, given an indel mutation candidate, we single out the MUA with the largest likelihood score and then draw a consensus sequence to represent the inserted or deleted consecutive bases. It should be noted that if two MUAs have an identical likelihood score, we consider the one with the more columns (i.e. the larger *l*) to have higher significance.

### Somatic variant calling

We call somatic variants from paired tumor-normal samples sequenced from tumor and normal tissues of the same individual, respectively. In this scenario, normal sample can act as a control in order to better distinguish variants that are unique to the tumor (somatic variants) from those present in the matched normal (germline polymorphisms).

#### Somatic SNV calling

In our algorithm, we have adopted a hybrid approach benefiting from the combination of an independent subtraction analysis and a joint sample analysis, with genotype inference as the core. Based on the genotypes inferred for both samples, SNVs detected in the tumor are classified into four mutation types: Somatic, LOH (loss of heterozygosity), Germline, and Unknown. Table 8 shows the type classification, where ${G^{N}_{k}}$ and ${G^{T}_{k}}$ (1≤*k*≤*K*) denote the genotypes from the normal and tumor, *A* and *B* denote the reference base *γ* and the non-reference variant (≠*γ*) in the diploid genotypes, respectively. Moreover, the SNVs are reported in the well-established VCF format [36].

**Table 8:** Type classification of somatic SNVs

${G^{N}_{k}}$\${G^{T}_{k}}$	AA	AB	BB
AA	Wild	Somatic	Somatic
AB	LOH	Germline	LOH
BB	Unknown	Unknown	Germline

##### Subtraction analysis

Our subtraction analysis first calls mutations from the normal and tumor samples separately using the aforementioned Bayesian probabilistic models and then contrasts the results like a simple subtraction. This approach can provide reasonable predictions if there exists little noise and variant alleles have large enough frequencies (e.g. exceeding the expected) to be detected. In SNVSniffer, given a site, the subtraction analysis works as follows : (*i*) call the genotype ${G^{N}_{k}}$ from the normal. If ${G^{N}_{k}}$ is not homozygous reference, the VCOS is computed for the genotype. ${G^{T}_{k}}$ is processed in the same way; and (*ii*) if ${G^{N}_{k}}$ is homozygous reference and ${G^{T}_{k}}$ has high-confidence variants, a Somatic-type SNV is reported for this site, and otherwise, leave it to the subsequent joint sample analysis.

##### Joint sample analysis

As mentioned above, somatic allele frequencies in the tumor may have considerable variability, often caused by the presence of normal cells in the tumor sample, copy number variations and tumor heterogeneity. In such cases, the simple subtraction analysis may become less effective since variant alleles might have considerably low frequencies compared to the expected frequencies. In this regard, a joint model to simultaneously analyze both samples will likely lead to an increased ability to detect shared signals, which arise from germline polymorphisms or sequencing cycles, as well as weakly observed real somatic variants. In SNVSniffer, this joint analysis is applied after the preceding subtraction analysis.

At each site *i* of the genome, we model allele counts of the tumor-normal pair as a joint multinomial conditional distribution, given a joint genotype ${G^{N}_{k}}$ and ${G^{T}_{t}}$, where 1≤*k*<*K* and 1≤*t*<*K*. Assume that *X*_*i*_ represents the allele count vector observed in the normal and *Y*_*i*_ the allele count vector observed in the tumor. The posterior probability $P({G^{N}_{k}}, {G^{T}_{t}} | X_{i}, Y_{i})$ of the joint genotype ${G^{N}_{k}}$ and ${G^{T}_{t}}$, given *X*_*i*_ and *Y*_*i*_, is computed as

14 $$ \begin{aligned} P\left({G^{N}_{k}}, {G^{T}_{t}} | X_{i}, Y_{i}\right)\propto P\left(X_{i}| Y_{i}, {G^{N}_{k}}, {G^{T}_{t}}\right)\\ \times P\left(Y_{i}| {G^{N}_{k}}, {G^{T}_{t}}\right) P\left({G^{N}_{k}}, {G^{T}_{t}}\right) \end{aligned}  $$

As mentioned above, the normal sample is assumed not to contain any read sequenced from tumor cells. This indicates that in our algorithm, *X*_*i*_ is independent of both *Y*_*i*_ and ${G^{T}_{t}}$. Hence, $P\left (X_{i} | Y_{i}, {G^{N}_{k}}, {G^{T}_{t}}\right)$ can be re-written as $P\left (X_{i} | {G^{N}_{k}}\right)$ and Eq. (14) can therefore be simplified as

15 $$ \begin{aligned} P\left({G^{N}_{k}}, {G^{T}_{t}} | X_{i}, Y_{i}\right)\propto P\left(X_{i}| {G^{N}_{k}}\right)\\ \times P\left(Y_{i} | {G^{N}_{k}}, {G^{T}_{t}}\right) P\left({G^{N}_{k}}, {G^{T}_{t}}\right) \end{aligned}  $$

where $P\left (X_{i} | {G^{N}_{k}}\right)$ is computed using Eq. (1). As for $P\left (Y_{i} | {G^{N}_{k}}, {G^{T}_{t}}\right)$, it is equal to $P\left (Y_{i} | {G^{T}_{t}}\right)$ if the tumor sample contains no normal cell (meaning that *Y*_*i*_ is independent of ${G^{N}_{k}}$), and thereby can be computed using Eq. (1). However, in practice, the tumor sample has a probability of incorporating normal cells and it would be more realistic to taken into account the tumor purity, which represents the expected percentage of alleles from tumor cells at each site, in our computation. For simplicity, our algorithm assumes $P\left (Y_{i} | {G^{N}_{k}}, {G^{T}_{t}}\right)$ equals $P\left (Y_{i} | {G^{T}_{t}}\right)$, but employs the tumor purity as an indicator to trade off sensitivity and specificity, especially at the sites with low variant fractions.

In Eq. (14), $P({G^{N}_{k}}, {G^{T}_{t}})$ can be re-written as $P({G^{N}_{k}}, {G^{T}_{t}}) = P({G^{T}_{t}} | {G^{N}_{k}})P({G^{N}_{k}})$. To compute $P({G^{N}_{k}}, {G^{T}_{t}})$, one approach is treating the genotypes of the two samples as completely independent events, where $P\left ({G^{N}_{k}}, {G^{T}_{t}}\right)$ can be computed as $P\left ({G^{N}_{k}}\right)P\left ({G^{T}_{t}}\right)$. Another approach is assuming that the genotypes of both samples are dependent. The latter is more realistic since the two samples are sequenced from the same individual and tend to share germline polymorphisms. In this regard, we have used the conditional probability $P\left ({G^{T}_{t}} | {G^{N}_{k}}\right)$ proposed in [16] by assuming that the genotypes of the two samples are dependent.

##### Post-processing procedure

Having calculated the most likely genotypes ${G^{N}_{k}}$ and ${G^{T}_{t}}$ using Eq. (14), our post-processing procedure consists of four steps: (*i*) if the new ${G^{N}_{k}}$ is not identical to the one called by the previous subtraction analysis, compute its VCOS if it is not homozygous reference. If the new ${G^{N}_{k}}$ has high-confidence variants, it is retained to replace the old one computed by the subtraction approach, and otherwise, ${G^{N}_{k}}$ is deemed as homozygous reference because of the lack of confidence. ${G^{T}_{t}}$ is processed in the same way; (*ii*) if the new ${G^{N}_{k}}$ is identical to the one called by the previous subtraction analysis, compute its VCOS with a relaxed constraint if it is not homozygous reference. This relaxed computation considers a genotype as non-false-positive if the number of variants in the genotype exceeds a minimum threshold conditioned on the read depth at the site. The new ${G^{T}_{t}}$ is also processed likewisely; (*iii*) if both ${G^{N}_{k}}$ and ${G^{T}_{t}}$ are classified as false positives, the called variant will be discarded and otherwise, retained; and (*iv*) classify the variant and report the result in VCF.

#### Somatic indel calling

For indel calling, our algorithm separately call mutations from the normal and tumor samples. At a given site, our algorithm will make an attempt to call a somatic indel if only one of the two samples is deemed as an indel candidate at the site. The consensus sequence of a somatic indel is determined using the MUA approach mentioned above.
